# Dyadic effects of perceived social support on family health and family functioning in patients with heart failure and their nearest relatives: Using the Actor–Partner Interdependence Mediation Model

**DOI:** 10.1371/journal.pone.0217970

**Published:** 2019-06-04

**Authors:** Mahdi Shamali, Hanne Konradsen, Lara Stas, Birte Østergaard

**Affiliations:** 1 Department of Clinical Research, University of Southern Denmark, Odense, Denmark; 2 Department of Gastroenterology, Herlev and Gentofte University Hospital, Copenhagen, Denmark; 3 Department of Neurobiology, Care Sciences and Society, Karolinska Instituttet, Huddinge, Sweden; 4 Department of Clinical Medicine, Faculty of Health and Medical Sciences, University of Copenhagen, Copenhagen, Denmark; 5 Department of Data Analysis, Ghent University, Gent, Belgium; 6 OPEN- Open Patient data Explorative Network, University of Southern Denmark, Odense, Denmark; Yale School of Medicine, UNITED STATES

## Abstract

**Background:**

Social support, family functioning and family health are essential elements in the treatment of heart failure, yet most heart failure studies focus on the pharmacological interventions. This study aimed to examine whether perceived social support from nurses is associated with better family functioning of patients with heart failure and their nearest relatives and to examine whether family health mediates this relationship.

**Methods and findings:**

A sample of 312 patients with heart failure and 312 of their nearest relatives were included in the study. The Family Functioning, Health and Social Support questionnaire was used to collect the data. Dyadic data were analysed by the Actor–Partner Interdependence Mediation Model with distinguishable dyads using structural equation modelling. Patients and nearest relatives who perceived more social support had a higher level of family health and functioned better within the family. One partner effect was found, indicating that the higher the level of family health of the nearest relative, the better the family functioning of the patient (p <0.001). Family health partially (in the patient) and completely (in the nearest relative) mediated the association between social support and family functioning.

**Conclusion:**

This study indicated that patients with heart failure and their nearest relatives who perceived more social support from nurses were more likely to have high level of family health and function better within the family. The interdependent relationships found in our study highlight a dyadic and family-oriented approach to improve family functioning in patients with heart failure.

## Introduction

Heart failure (HF), a major clinical and public health problem, is becoming a leading cause of disability and death among older adults [1–3]. In developed countries, 1–2% of the general population has HF [3], and the 5-year mortality rate among patients with HF is similar to that of many cancers [4]. Rehospitalisation is common among patients with HF, with more than 50% readmission within 6 months of discharge [5]. HF is a burden on health care services due to the significant mortality, morbidity, hospitalizations and health care expenditures associated with it [1, 3, 4]. HF can also be perceived as a burden on the family due to disagreement between patients and their family members on illness management [6, 7].

During the treatment of HF, patients and their families are affected by the patients’ health status [8]. Family health is considered as a unit of health involving each member of the family and the family as a whole [9]. It includes well-being, ill-being, values, knowledge, and healthy activities [10]. The severity of illness is negatively associated with family functioning. It has shown that in more severe illness, families are more at risk for low cohesion and increased conflict [11]. Hence, healthy individuals function better within a family. Family health and family functioning play important roles in self-care behaviours, medication adherence, mortality and morbidity in patients with HF [12] through the lifestyle modifications of the family to the illness [13]. Family functioning and family health are essential elements in the treatment of HF. Nonetheless, most HF studies focus on the pharmacological interventions [14] without considering the role of the partner or family in this disease [15].

In addition, social support has been considered an important resource to improve an individual’s family health and family functioning [16, 17]. Social support improves an individual’s psychological well-being (e.g., decreases depression and anxiety) and physical health (e.g., increases healthy activities and protective behaviours and promotes a healthier lifestyle); accordingly, the individual functions better within the family [18]. A few studies conducted among the families of patients with heart disease have indicated that the more social support received from nurses the better the family functioned [16, 17, 19]. These assessments have not been evaluated with respect to the interdependence between patients with HF and their partners at the dyadic level. However, studies emphasized the need for more research to address the lack of specific attention to the study of dyadic analysis in HF [8, 20].

In interdependence theory, interactions between individuals in a close relationship influence their respective partners’ outcomes. The Actor–Partner Interdependence Model (APIM) provides accurate estimates of both individual and dyadic effects by modelling the influence of dyad members on each other [21]. The APIM can estimate to what extent the independent variable of a person (e.g., the patient’s social support) influences his/her own score on the dependent variable (e.g., the patient’s family functioning), denoted as the actor effect, as well as on the dependent variable of his/her partner (e.g., the nearest relative’s family functioning), denoted as the partner effect [22]. An extended model of the APIM, the Actor–Partner Interdependence Mediation Model (APIMeM), has been suggested for use to assess mediation in dyadic data [23].

Despite the important role of family functioning and family health in the treatment of HF, the influence of social support on family health and family functioning, and inevitable influential interactions between the patient–nearest relative dyad in HF, no study of dyadic analysis that explores the associations among social support, family functioning, and family health following HF has been reported. Therefore, there is a need for studies that contribute to a better understanding of the intra-dyadic associations between social support, family health and family functioning in patients with HF and their relatives. We assume that social support is directly and/or indirectly (through family health) associated with family functioning. Hence, the aim of this study was to examine whether perceived social support from nurses in patients with HF and their nearest relatives is associated with their own and their nearest relatives’/patients’ family functioning and to examine whether family health in patients with HF and their nearest relatives mediates this relationship.

## Methods

### Study design, participants, study setting and data collection

Using a cross-sectional study design, this was a secondary analysis of the baseline data of a randomized multicentre clinical trial. The original study aimed to investigate the effect of family health conversations among outpatients with HF and their nearest relatives in a sample of 790 individuals (468 patients with HF and 322 nearest relatives) who were asked to complete the Family Functioning, Health and Social Support (FAFHES) questionnaire. The study received ethical approval from the local research ethics committee of the Region of Southern Denmark (No: s-20110068) and the Danish Data Protection Agency (J.nr.2012-54-0140) and was registered in clinicaltrials.gov (NCT01378247). It was conducted from June 2011 to January 2017. The participants, consisting of patients and their nearest relatives who met the inclusion criteria, were consecutively enrolled in the study and completed separate copies of the questionnaire. Patients from three Danish HF outpatient clinics who were confirmed to have an HF diagnosis, had left ventricular ejection fraction (LVEF) ≤ 40%, had New York Heart Association classification (NYHA) II-IV symptoms and had signed informed consent along with their nearest relatives were included in the study. Patients who were in the terminal stage of other serious diseases with a life expectancy of less than 6 months were excluded from the study.

We assessed the baseline data of 468 patients with HF and 322 nearest relatives from the original study for eligibility to be included as a dyad (patient with his/her nearest relative) in the present study. We identified 320 dyads to be eligible, of whom eight dyads were excluded because of incomplete data. Finally, we included the baseline data of 312 Danish patient–nearest relative dyads (a total of 624 subjects) with no missing data from January 2015 to January 2016. The current study was approved by the Danish Data Protection Agency (J.nr. 18/24435). Formal ethical permission was not required according to Danish legislation, because this was a secondary analysis of the ethically approved study.

### Instrument

The FAFHES questionnaire was used to collect the data [10]. The FAFHES was developed and tested for the study of families of heart patients by measuring the support that families receive in different life situations and its impact on family functioning and health [10, 24]. The FAFHES questionnaire consists of three scales and 62 items: family functioning (19 items), family health (23 items) and social support (20 items). The sub-areas of family functioning, family health and social support scales are shown in Table 1. The scale is a 6-point Likert scale ranging from 1 (strongly disagree) to 6 (strongly agree). Higher scores represent better functioning. The questionnaire also includes the dyad’s demographic characteristics (e.g., gender, age, marital status, education, work status and relationship of the nearest relative to patient) and patient clinical characteristics (e.g., NYHA classification, length of illness, comorbidity, and LVEF).

**Table 1:** FAFHES questionnaire’s subscales and descriptions.

Scale	Number of Items	Description
**Family functioning**	19	
Family relationships	7	Emotional ties and shared experience
Relationships outside the family	5	Finding mental support outside the family
Structural factors of family	4	Family structure in planning, working as a team and sharing experiences
Strengths of family	3	Resources inside and outside the family
**Family health**	23	
Values	6	Such as freedom, peace, security, integrity, and humour
Well-being	4	Such as effortless coping and feelings of energy
Knowledge	5	Knowledge of one’s own and others’ health, health problems, possible solutions, and sources of help
Ill-being	5	Feelings of discomfort and bad feelings
Activities	3	Person’s healthy activities
**Social support**	20	
Affect	8	Emotional support (e.g., offering of empathy, concern, affection, love, trust, acceptance, intimacy, encouragement, or caring)
Affirmation	7	Informational support, support for decision making, appreciation and admiration
Concrete aid	5	Instrumental help, time spent helping someone & services

The reliability of the questionnaire measured using Cronbach’s alpha varied from 0.77 to 0.88 for family functioning, from 0.73 to 0.86 for family health and from 0.92 to 0.95 for social support, showing acceptable to excellent reliability [10, 19]. The reliability of the questionnaire measured among patients with HF in the Danish population using Cronbach’s alpha ranged from 0.73 to 0.95 across the three scales, and test-retest reliability using interclass correlation coefficients ranged from 0.69 to 0.86, indicating acceptable to excellent reliability [25].

### Data analysis

Descriptive statistics, such as frequency distributions or means and standard deviations, were obtained using Stata statistical software version 15.0 (StataCorp LLC, College Station, TX) to summarize demographic and clinical characteristics. As proposed by Ledermann et al. [23], an APIMeM for distinguishable dyads (the dyad members were considered to be distinguishable based on HF diagnosis) with mixed variables was conducted to examine the effect of social support (predictor variable) on family functioning (outcome variable) through family health (mediating variable). In the APIMeM, the effect of social support on family health is designated as *a*, the effect of family health on family functioning is designated as *b*, and the effect of social support on family functioning is designated as *c’*. Then, the direct effects (*a*, *b* & *c’*), the mediating or indirect effects (*ab*) and the total effects (*ab+c’*) were computed (Table 2). The indirect effect is estimated as the direct effect *a* times *b* and reflects how much of the association between social support and family functioning is explained by family health. The total effect is the sum of the direct effect *c’* and the indirect effect *ab* and represents the relationship between social support and family functioning before adjustment for family health. The APIMeM analysis was performed in AMOS 22 and Stata statistical software 15 using structural equation modelling with a dyadic dataset. We used the bias-corrected 95% CI bootstrap sample of 5000 by means of Monte Carlo sampling to test the significance of the mediating and total effects. The overall equation-level goodness of fit for APIMeM was evaluated using R-squared (*R*^*2*^*)*. Finally, a graphical representation of the mediated effect for both the patients and the nearest relatives is displayed using the R package, as proposed by Fritz and MacKinnon [26]. All analyses were two‑tailed, and a *p*-value < 0.05 was considered statistically significant in all tests.

**Table 2:** The total effects, total indirect effects, simple indirect effects, and direct effects in the APIMeM in patients with heart failure and their nearest relatives.

Effect		Coefficient	Label
**Actor effect** (Individual’s perceived social support→Individual’s family functioning)			
Patient	Total effect	*a*_*A*1_*b*_*A*1_ + *a*_*P*2_*b*_*P*1_ + *c*′_*A*1_	Patient actor total effect
	Total IE	*a*_*A*1_*b*_*A*1_ + *a*_*P*2_*b*_*P*1_	Patient actor total IE
	Actor–actor simple IE	*a*_*A*1_*b*_*A*1_	Patient actor–actor IE
	Partner–partner simple IE	*a*_*P*2_*b*_*P*1_	Patient partner–partner IE
	Direct effect	*c*′_*A*1_	Patient actor direct effect
Nearest relative	Total effect	*a*_*A*2_*b*_*A*2_ + *a*_*P*1_*b*_*P*2_ + *c*′_*A*2_	Nearest relative actor total effect
	Total IE	*a*_*A*2_*b*_*A*2_ + *a*_*P*1_*b*_*P*2_	Nearest relative actor total IE
	Actor–actor simple IE	*a*_*A*2_*b*_*A*2_	Nearest relative actor–actor IE
	Partner–partner simple IE	*a*_*P*1_*b*_*P*2_	Nearest relative partner–partner IE
	Direct effect	*c*′_*A*2_	Nearest relative actor direct effect
**Partner effect** (Individual’s perceived social support→Partner’s family functioning)			
Patient	Total effect	*a*_*A*2_*b*_*P*1_ + *a*_*P*1_*b*_*A*1_ + *c*′_*P*1_	Patient partner total effect
	Total IE	*a*_*A*2_*b*_*P*1_ + *a*_*P*1_*b*_*A*1_	Patient partner total IE
	Actor–partner simple IE	*a*_*A*2_*b*_*P*1_	Patient actor–partner IE
	Partner–actor simple IE	*a*_*P*1_*b*_*A*1_	Patient partner–actor IE
	Direct effect	*c*′_*P*1_	Patient partner direct effect
Nearest relative	Total effect	*a*_*A*1_*b*_*P*2_ + *a*_*P*2_*b*_*A*2_ + *c*′_*P*2_	Nearest relative partner total effect
	Total IE	*a*_*A*1_*b*_*P*2_ + *a*_*P*2_*b*_*A*2_	Nearest relative partner total IE
	Actor–partner simple IE	*a*_*A*1_*b*_*P*2_	Nearest relative actor–partner IE
	Partner–actor simple IE	*a*_*P*2_*b*_*A*2_	Nearest relative partner–actor IE
	Direct effect	*c*′_*P*2_	Nearest relative partner direct effect

Note: APIMeM, actor–partner interdependence mediation model; *a*, direct effect of perceived social support on family health; *b*, direct effect of family health on family functioning; *c*′, direct effect of perceived social support on family functioning; *A*, actor effect; *P*, partner effect; IE, indirect effect; 1, patient; 2, nearest relative.

In the present study, the power analysis was based on the multiple regression analysis, as recommended by Kenny and Cook [27], when structural equation modelling is used in dyadic analyses without latent variables. Statistical power analysis was performed in the G*Power 3.1.9.2 program [28] using linear multiple regression. With a sample size of 312 dyads in our study, 12 predictors and α = 0.05, we had 95% power to detect a small effect (*f* ^*2*^ = 0.09).

## Results

### Sample characteristics

In the present study, 312 patient–nearest relative dyads were included and analysed with no missing data. The sample characteristics are shown in Table 3. The mean age of the dyads was 64.7 years for patients and 58.9 years for nearest relatives. Most patients were male (71.2%), and most of the nearest relatives were female (66.3%). The majority of the dyads were married, 68.6% of patients and 58.7% of nearest relatives. The education level for the highest percentage was the high school level, 35.9% of patients and 37.2% of nearest relatives. Almost 60% of the patients and 46% of the nearest relatives were retired. Most patients had NYHA Class II HF with 80.1%, and the mean duration of HF was 4.4 months with a median of 1 month. The mean LVEF was 28.4%. Hypertension (38.5%) and myocardial infarction (37.8%) were the most common comorbid conditions in the patients. Most of the nearest relatives (69.2%) were the spouse/partner of the patients. Patients had higher mean scores in family functioning, family health and perceived social support compared with the nearest relatives (Table 3).

**Table 3:** Characteristics of patient–nearest relative dyads.

Characteristics	Patients (n = 312) M ± SD or n (%)	Nearest Relative (n = 312) M ± SD or n (%)
Sex, female	90 (28.8)	207 (66.3)
Age, years	64.7 ± 12.4	58.9 ± 15.6
Marital status		
Married	214 (68.6)	183 (58.7)
Cohabiting	36 (11.5)	49 (15.7)
Single	29 (9.3)	47 (15.1)
Divorced	22 (7.1)	20 (6.4)
Widower	11 (3.5)	13 (4.2)
Education		
Elementary school	64 (20.5)	67 (21.5)
High school	112 (35.9)	116 (37.2)
College/ Bachelor	93 (29.8)	96 (30.8)
Higher education	43 (13.8)	33 (10.6)
Work status		
Self-employed	8 (2.6)	23 (7.4)
Employee	101 (32.4)	137 (43.9)
Unemployed	15 (4.8)	8 (2.6)
Retired	187 (59.9)	144 (46.2)
NYHA classification		
NYHA II	250 (80.1)	
NYHA III	59 (18.9)	
NYHA IV	2 (0.6)	
Duration of HF, months	4.40 ± 10.35 [1 (1–3)]^a^	
Comorbidity		
Diabetes	52 (16.7)	
Hypertension	120 (38.5)	
Stroke	35 (11.2)	
Atrial fibrillation	95 (30.4)	
Myocardial infarction	118 (37.8)	
COPD	48 (15.4)	
LVEF, %	28.4 ± 3.7	
Relation to patient		
Spouse / Partner		216 (69.2)
Child		51 (16.3)
Parent		11 (3.5)
Sister/brother		16 (5.1)
Daughter in law/son in law		9 (2.9)
Others		9 (2.9)
Perceived social support	83.1 ± 22	78.7 ± 22.2
Family functioning	90.6 ± 13.8	89.6 ± 13.8
Family health	108.7 ± 11.4	106. 9 ± 11.9

Note: M, mean; SD, standard deviation; NYHA, New York Heart Association Classification; COPD, chronic obstructive pulmonary disease; LVEF, left ventricle ejection fraction.

^a^ Median and interquartile range (25% to 75%)

### Actor–Partner Interdependence Mediation Model analysis

The residuals of the regression were normally distributed. A few mild outliers were detected, which were legitimate observations. Data analyses were performed while keeping these outliers. The test of empirical distinguishability (which includes constraining six actor and partner effects to be equal for both roles across dyads in the APIMeM and then testing the goodness of fit for the model) was statistically significant [*χ*^*2*^ (6) = 14.23, *p* = 0.027, RMSEA = 0.066], indicating that members can be statistically distinguished based on the HF diagnosis. The overall equation-level goodness of fit for APIMeM was *R*^*2*^ = 0.17, indicating that 17% of the variations in family health and family functioning are explained by the model.

#### Direct actor and partner effects

**Fig 1:**
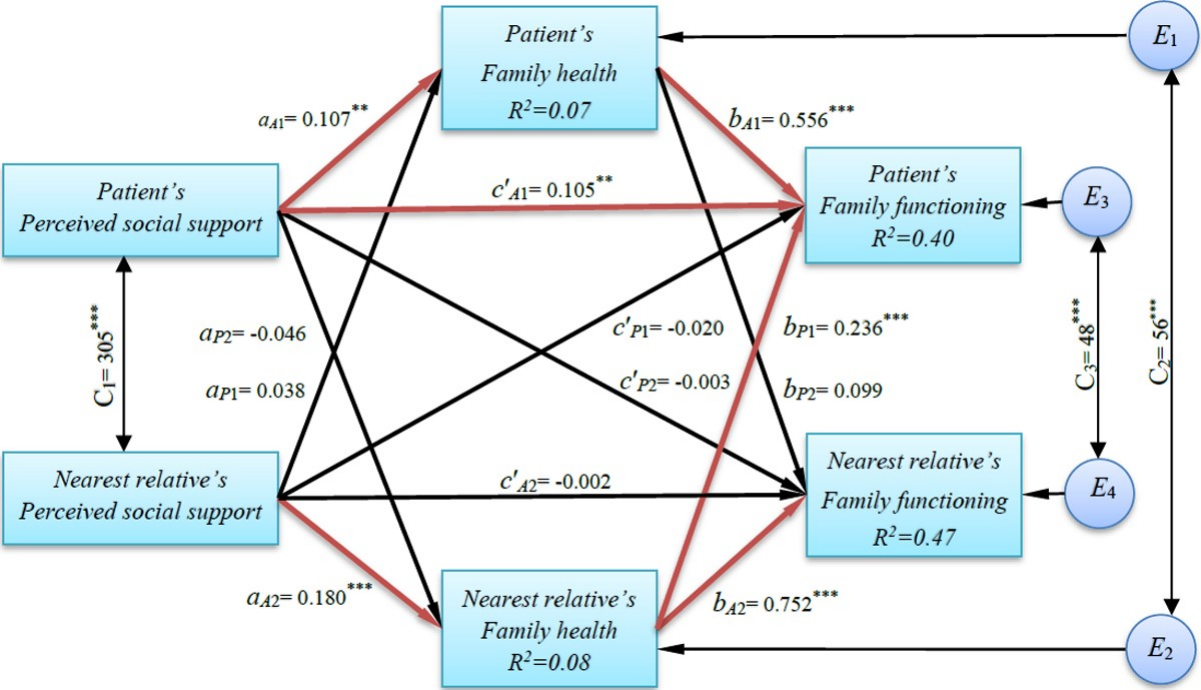
The Actor–Partner Interdependence Mediation Model in patients with heart failure and their nearest relatives. Note. *a*, direct effect of perceived social support on family health; *b*, direct effect of family health on family functioning; *c*′, direct effect of perceived social support on family functioning; *A*, actor effect; *P*, partner effect; C_1_, covariance between the two predictor variables; C_2_ & C_3_, covariance between the two error terms; E_1_, E_2_, E_3_ & E_4_, latent error terms; R^2^, coefficient of determination; 1, patient; 2, nearest relative; Estimates are unstandardized regression coefficients; Significant path coefficients are in red. ** p < .01; *** p < .001.

The direct effect of social support on family functioning emerged as a significant actor effect for patients (*B* = 0.105, *p* = 0.004), indicating that the patients’ own social support was positively associated with their own family functioning. That is, patients who perceived more social support also functioned better within the family. In contrast, the actor effect for nearest relatives was not significant (*B* = −0.002, *p* = 0.944; Table 4 and Fig 1). Moreover, the partner effect from patient to nearest relative (*B* = −0.003, *p* = 0.938) and the partner effect from nearest relative to patient were not statistically significant (*B* = −0.020, *p* = 0.596; Table 4 and Fig 1). That is, no association was found between an individual’s partner’s perceived social support and the individual’s own family functioning.

**Table 4:** The total effects, total indirect effects, and direct effects in the APIMeM in patients with heart failure (n = 312) and their nearest relatives (n = 312).

Effect		*B*	SE	95% CI	*p*-value
**Actor effect**					
Patient	Total effect	0.153	0.043	0.068, 0.240	< .001
	Total IE	0.048	0.027	−0.004, 0.101	0.069
	Direct effect	0.105	0.036	0.034, 0.175	0.004
Nearest relative	Total effect	0.137	0.044	0.050, 0.224	0.002
	Total IE	0.139	0.031	0.078, 0.200	< .001
	Direct effect	−0.002	0.034	−0.069, 0.064	0.944
**Partner effect**					
Patient	Total effect	0.044	0.042	−0.040, 0.128	0.300
	Total IE	0.063	0.027	0.010, 0.116	0.019
	Direct effect	−0.020	0.036	−0.090, 0.052	0.596
Nearest relative	Total effect	−0.026	0.045	−0.114, 0.061	0.555
	Total IE	−0.024	0.031	−0.085, 0.037	0.444
	Direct effect	−0.003	0.034	−0.069, 0.064	0.938

Note: APIMeM, actor–partner interdependence mediation model; *B*, unstandardized regression coefficients; SE, standard error; CI, confidence interval; IE, indirect effect.

The direct effect of social support on family health emerged as a significant actor effect for patients (*B* = 0.107, *p* = 0.003) and a significant actor effect for nearest relatives (*B* = 0.180, *p* < 0.001; Fig 1), indicating that for both the patients and the nearest relatives, their own perceived social support was positively associated with their own family health. In contrast, the partner effect from patient to nearest relative (*B* = –0.046, *p* = 0.225) and the partner effect from nearest relative to patient were not statistically significant (*B* = 0.038, *p* = 0.296; Fig 1). That is, no association was found between an individual’s or partner’s perceived social support and the individual’s own family health.

The direct effect of family health on family functioning emerged as a significant actor effect for patients (*B* = 0.556, *p* < 0.001) and a significant actor effect for nearest relatives (*B* = 0.752, *p* < 0.001; Fig 1), indicating that for both the patients and the nearest relatives, their own family health was positively associated with their own family functioning. Furthermore, the partner effect from the nearest relative to the patient was statistically significant (*B* = 0.236, *p* < 0.001). That is, the higher the level of family health in the nearest relative, the better the patient’s family functioning. However, the partner effect from patient to nearest relative was not statistically significant (*B* = 0.099, *p* = 0.084; Fig 1).

#### Indirect and total effects of social support on family functioning

**Fig 2:**
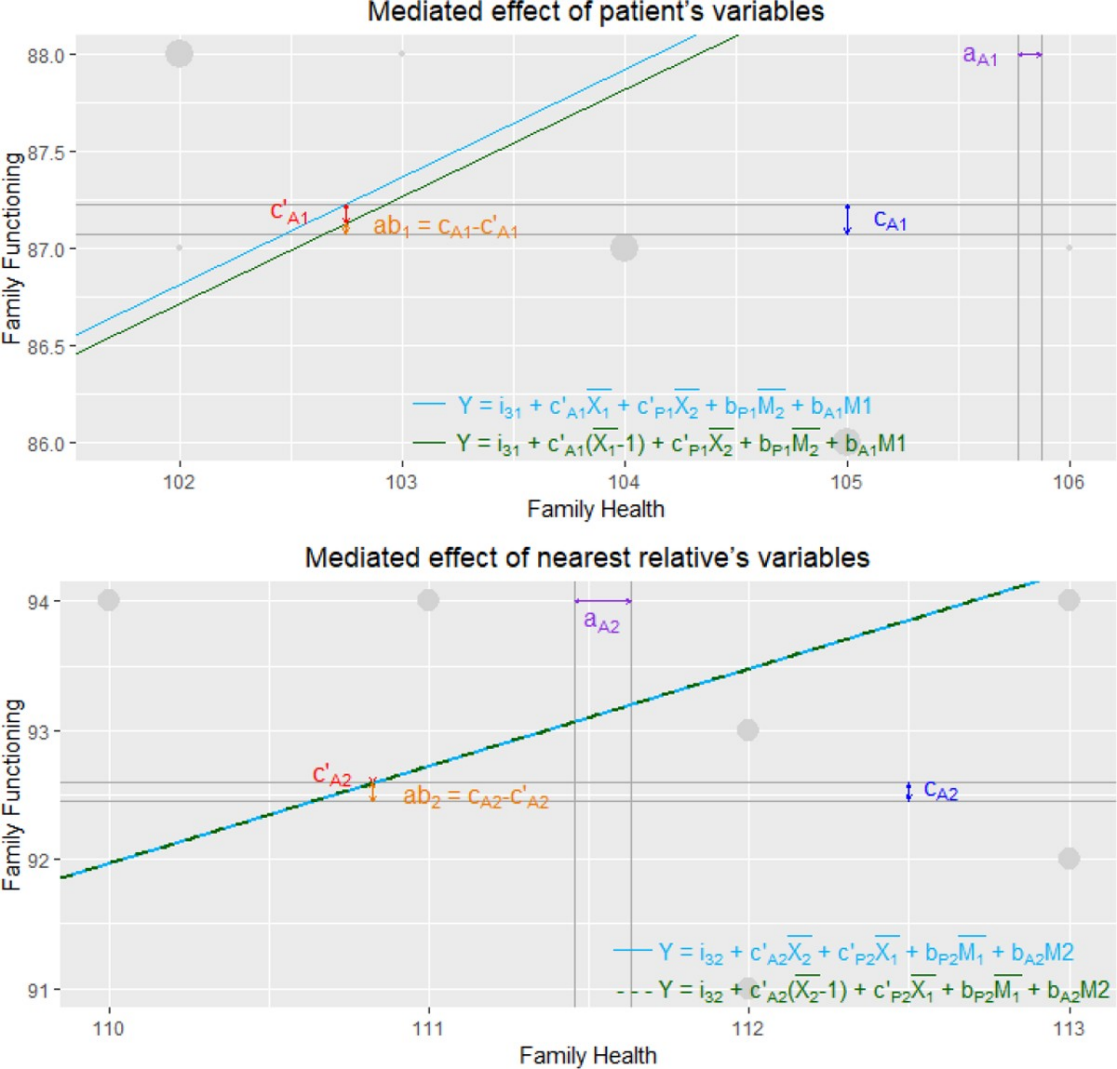
A graphical representation of the mediated effect for patients and nearest relatives. For clarity reasons, only a small section of the overall graph is presented. Dots represent participants scores, with larger dots representing more observations. The distance between the vertical lines (i.e., a_A1_ and a_A2_) represents the predicted unit change in family health for a one unit change in social support for patients and nearest relatives, respectively. The distance between the horizontal lines (i.e., c_A1_ and c_A2_) depicts the predicted unit change in family functioning for a one unit change in social support. The distance between the two regression lines (i.e., c’_A1_ and c’_A2_) outlines the predicted unit change in family functioning for a one unit change in social support when holding the other mediating and predictor variables constant. The slope of the regression lines is the predicted unit change in family functioning for a one unit change in family health, adjusted for social support of both roles and family health of the other role. Finally, the indirect effect (i.e., ab_1_ and ab_2_) is the difference between the total effect (i.e., c_A1_ and c_A2_, respectively) and the direct effect (i.e., c’_A1_ and c’_A2_, respectively). Note. a (aA1/aA2), effect of perceived social support on family health; c (cA1/cA2), total effect of perceived social support on family functioning; ab, indirect effect of perceived social support on family functioning; c’, direct effect of perceived social support on family functioning. Blue and red slopes indicate effect of family health on family functioning.

The mediation analyses with patient variables showed that both estimated paths (social support→family health→family functioning) for the indirect effect (actor–actor effect) and the direct effect from social support to family functioning were statistically significant (Fig 1), indicating that family health partially mediated the relationship between perceived social support from nurses and family functioning in patients with HF (explaining 36.3% of the total effect). The mediation analyses with nearest relative variables showed that both estimated paths (social support→family health→family functioning) for the indirect effect (actor–actor effect) were statistically significant, while the estimate of the direct effect from social support to family functioning was not significant (Fig 1). Therefore, family health completely mediated the relationship between perceived social support from nurses and family functioning in nearest relatives (explaining 98.5% of the total effect). Fig 2 illustrates the plots of the mediated effect of patient’s and nearest relative’s variables. Looking at the plots, the length of *ab* relative to the length of *c* (i.e., c_A1_/c_A2_) indicates the amount of the overall effect of perceived social support on family functioning mediated by family health; it accounts for 36.3% and 98.5% of the total effect for the patients and for the nearest relatives, respectively.

The mediation analyses of the partner effects showed that only the paths (nearest relative’s perceived social support→nearest relative’s family health→patient’s family functioning) for the patient indirect actor–partner effect were statistically significant (Fig 1). That is, the nearest relative’s family health completely mediated the relationship between the nearest relative’s perceived social support and the patient’s family functioning (explaining 98.2% of the total effect).

Moreover, the total actor effect for the patient (*B* = 0.153, *p* < 0.001) and the total actor effect for the nearest relative (*B* = 0.137, *p* = 0.002) were statistically significant (Table 4). The total partner effect for the patient (*B* = 0.044, *p* = 0.3) and the total partner effect for the nearest relative (*B* = –0.026, *p* = 0.555) were not statistically significant (Table 4).

## Discussion

In this dyadic analysis of a sample of patients with HF and their nearest relatives, 5 actor effects (of 6 possible), 1 partner effect (of 6 possible), 2 indirect actor effects (of 2 possible) and 1 indirect partner effect (of 4 possible) were identified. Similarly, previous studies using dyadic analysis in a cardiovascular context indicated a larger number of actor effects than partner effects [8, 20, 29].

In the current study, a high level of perceived social support from nurses was associated with a higher level of family health and better family functioning in patients with HF and their nearest relatives. Similar results are reported in studies conducted among the family members of patients with heart disease [16, 17, 19]. Two studies indicated that greater social support from nurses is associated with better family functioning [16, 17]. Another study reported that high levels of family health are associated with high levels of family functioning in family members of heart disease patients [19].

In this study, a patient indirect actor–partner effect was found, indicating that perceived social support to nearest relatives was associated indirectly with both their own and the patients’ family functioning by enhancing their own family health. That is, the more perceived social support given the nearest relatives, the better the family functioning of patients. This finding highlights the interdependent relationships between nearest relatives and patients with HF that influence the patient’s outcomes, suggesting the need for a dyadic and family-oriented approach (including both the patient and his/her family member in the patient’s care) to improve family functioning. The research to date has tended to focus on the patient or relative separately rather than considering the patient and his/her relative as a unit of analysis. The dyadic approach is also recommended in previous studies for use in studies that address quality of life [8, 20, 30] and self-care management [6] in patients with HF. An interdependent relationship was found by Al-Rawashdeh et al. [30], who reported that HF patients’ mental well-being is associated with their spouses’ sleep disturbances. Another study by Buck et al. [6] also found an interdependent relationship between patients with HF and their caregivers, where the high level of caregiver depression was associated with lower HF patient self-care maintenance scores.

In our study, the total association between social support and family functioning was partially (in the patient) and completely (in the nearest relative) mediated by family health. The mediation effect of family health may be attributed to the close relationships between the subareas of family health and family functioning. For instance, perceived social support from nurses may have enhanced health knowledge, well-being and healthy activities (subareas of family health) and consequently led to improved relationships inside and outside the family and strengthened the family structure (subareas of family functioning). A serious weakness with this argument is, however, that there is neither an exact theoretical framework nor empirical evidence to explain the mediating effect of family health in previous studies, particularly at the dyadic level. To our knowledge, this is the first study to use APIMeM in a study of the associations between social support, family health and family functioning and to identify actor, partner and mediating effects. No other studies comparing social support, family health and family functioning in patients with HF and their relatives have been identified, therefore limiting empirical evidence for population comparisons. The current study may contribute to the empirical evidence needed to develop a theory in this regard. However, a longitudinal study is needed to clarify the association between social support, family health and family functioning.

## Implications

Based on the results of this study, patients with HF and their nearest relatives who had more perceived social support tended to have higher levels of family health and family functioning. One possible implication of this is that health care providers should consider incorporating social support for HF patients and their relatives to enhance family health and family functioning. Family functioning is important in modifying the lifestyle of the patient and family in response to an illness [31]. Hence, by improving family functioning, patients with HF and their families can better cope with the disease. Family functioning is also a nonpharmacological approach, by promoting self-care behaviours and medication adherence, to improve the outcomes in patients with HF [12]. Interventional studies are needed to better address the association between social support and family functioning.

Furthermore, the interdependent association between patients with HF and their nearest relatives’ outcomes found in our study highlights the importance of dyad- and family-focused approaches in the clinical setting and research. The APIM can be a good model to use in family-focused studies in patients with HF because it addresses inclusion of both the patient and his/her partner in the study. This study may also contribute to a better understanding of family dynamics in HF and may have implications for future research on family functioning in HF patients.

## Strengths and limitations

The major strength of this study is the analytic method, which used the APIMeM to detect actor, partner and mediating effects. The inclusion of both patients and their nearest relatives was another strength of this study. However, the findings of this study must be considered with some caveats. First, this was a cross-sectional study without a control group and thus did not allow any inferences of causality in these relationships. Second, our findings may reflect a selection bias as patients and their relatives experiencing low levels of family health and family functioning may have had less motivation to participate in the study. Third, some patients and their relatives might have special challenges in their lives (not related to HF) that might impact their family health and family functioning. However, we did not assess such challenges. Finally, the results of our study can only be applied to the type of patient and nearest relative we studied. In our study, most of dyads were married, most of nearest relatives were spouses, and most of patients were newly diagnosed with HF (median of 1 month) and had mild HF symptoms (NYHA Class II). Therefore, our results should be generalized with caution to other types of patients and their nearest relatives.

## Conclusions

This study indicated that patients with HF and their nearest relatives who perceived more social support from nurses were more likely to have a high level of family health and function better within the family. An interdependent relationship between patients with HF and their nearest relatives (dyadic effect) was found in this study, where a greater amount of perceived social support in nearest relatives was related to better family functioning in patients. Based on these findings, patients with HF will possibly benefit from dyad- and family-focused approaches that include both the patient and his/her relatives to improve family health and family functioning. Family health partially (in the patient) and completely (in the nearest relative) mediated the association between social support and family functioning. Research is needed to explore this mediational association.
